# Application of the pancreatic body suspension technique in laparoscopic splenectomy combined with selective pericardial varicosity dissection: An observational study

**DOI:** 10.1097/MD.0000000000039618

**Published:** 2024-09-06

**Authors:** Daohai Qian, Bin Liu, Bin Jiang, Shihang Xi, Xu Wang, Xiaoming Wang

**Affiliations:** a Department of Hepatobiliary Surgery, The First Affiliated Hospital of Wannan Medical College, Wuhu, Anhui, P.R. China.

**Keywords:** cirrhosis, esophagogastric fundal varicosity, hypersplenism, laparoscopic splenectomy combined with pericardial dissection, pancreatic body suspension

## Abstract

To investigate the safety of pancreatic body suspension (PBS) technique in laparoscopic splenectomy combined with pericardial devascularization for patients. A retrospective study inclusive of 16 patients who underwent laparoscopic splenectomy combined with pericardial devascularization from 2017 to 2022 was performed. A total of 5 patients underwent PBS technique and 11 underwent the traditional technique. There was no significant difference in age, sex, body mass index (BMI), preoperative serum white cell count (WBC), platelets (PLT), hemoglobin (HB), albumin (ALB), prothrombin time (PT), total bilirubin (TBIL), or spleen size between the 2 groups (*P* > .05). In the PBS group, the operation time was 280 minutes. The estimated intraoperative blood loss (EBL) was 250 mL. The mean postoperative hospitalization length was 11.2 days. There was no conversion to an open procedure or postoperative bleeding. In the traditional method group, the mean operation time was 240.91 minutes. The EBL was 290.91 mL. There were 2 cases of conversion to open, 3 cases of postoperative bleeding, and 1 reoperation. The incidence of postoperative short-term complications (postoperative bleeding, reoperation) was significantly higher in the traditional method group than in the PBS group (36.36% vs 0%, *P* = .034). PBS technique improved the safety of laparoscopic splenectomy combined with pericardial dissection and is worthy of clinical promotion.

## 1. Introduction

Hepatic cirrhosis is relatively common and is related to hepatitis B and C, alcohol and other factors. It mainly manifests as portal hypertension, splenomegaly, hypoproteinemia, ascites, and esophagogastric fundal varices.^[1,2]^ From initial compensatory cirrhosis to life-threatening decompensated cirrhosis, the pathological process of cirrhosis is irreversible. The most definitive treatment is liver transplantation^[3]^; however, due to the lack of donor livers, many patients with end-stage cirrhosis die while waiting for a liver.^[3]^ Rupture and hemorrhage of esophageal and gastric varices are the main causes of death.^[4]^ In addition, patients with cirrhosis have poor coagulation function. Coagulation function is further worsened when combined with hypersplenism and thrombocytopenia increasing the risk of spontaneous bleeding. Pericardial vascular dissection combined with splenectomy can effectively control massive gastrointestinal tract bleeding and also correct thrombocytopenia. In addition, to preserve gastric vascular and vagus nerve function, selective esophagogastric fundal vascular dissection is commonly used.^[5,6]^

Although open splenectomy combined with pericardial dissection has been previously performed, its serious postoperative abdominal adhesions markedly increase the technical difficulty and risk of complications during future liver transplantation. Therefore, in recent years, many clinicians recommend the laparoscopic approach.^[7]^ It has even been used in elderly patients,^[8]^ massive hypersplenism^[9]^ and large varicose veins.^[10]^ Although it has advantages over the open approach, laparoscopic splenectomy combined with pericardial dissection is a relatively difficult operation and a challenge for surgeons. The main reason is the risk of rupture of the varicosities causing massive bleeding. Patients with hypersplenism and thrombocytopenia are more likely to experience massive life endangering intraoperative hemorrhage. Thus, the conversion rate to a laparotomy reaches 13%.^[11]^ Therefore, the technique of a safe laparoscopic splenectomy combined with pericardial dissection is an important surgical topic. In our early clinical practice, we explored the pancreatic body suspension (PBS) technique. Recently, laparoscopic selective pericardial devascularization has been demonstrated to be superior to nonselective pericardial devascularization in terms of preserving the gastric blood supply.^[6]^ Therefore, we adopted the technique of laparoscopic splenectomy combined with selective pericardial dissection and achieved good results as reported below.

## 2. Materials and methods

### 2.1. Patients and study design

A retrospective analysis was performed on 16 patients who underwent laparoscopic splenectomy combined with selective pericardial devascularization in our department from 2017 to 2022. Five cases were performed using the PBS technique and 11 were performed using the traditional method. Informed consent was obtained from all patients. Clinical data including sex, age, weight-to-height ratio, American College of Anesthesiologists (ASA) score, Child–Pugh grade and etiology of cirrhosis were recorded. Operative factors including biochemical indexes, operation duration, conversion to laparotomy and intraoperative blood loss were also recorded. Outcomes including postoperative pancreatic fistula, bleeding, postoperative hospitalization days and requirement for reoperation were collected and analyzed.

### 2.2. Inclusion and exclusion criteria

Each patient was required to demonstrate the following for study inclusion: hepatic cirrhosis diagnosed by clinical signs, imaging and laboratory examination; splenic enlargement with platelet count below 70,000 per milliliter of blood; no known organic disease(s); the ability to tolerate surgery; good compliance; no obvious abdominal adhesions; and a Child–Pugh score ≤ 10 points. Gastroscopy was used to evaluate varicosities in the esophagogastric fundus with a red sign or if the patient had a history of hematemesis and had previously undergone variceal ligation.

Exclusion criteria were: an ASA score > 3 points, patients over 80 years old, poor compliance and severe abdominal adhesions based on intraoperative diagnosis.

### 2.3. Statement of ethics

All patients and/or their legal guardian(s) were informed of the intraoperative anastomosis methods, operation risk, etc, and signed the informed consent form for operation. The study was conducted according to the guidelines laid down in the Declaration of Helsinki and approved by the Research Ethics Committee of the First Affiliated Hospital of Wannan Medical College (2018-13), and the requirement to obtain informed written consent was waived.

### 2.4. Statistical analysis

Data are expressed by means ± standard deviation (X ± SD). The *t*-test and rank sum tests were used for comparison between continuous variables. The χ^2^ test was used to determine differences between categorical variables. SPSS 22.0 was used for statistical analysis.

## 3. Surgical method

### 3.1. The traditional approach

**Figure 1.**
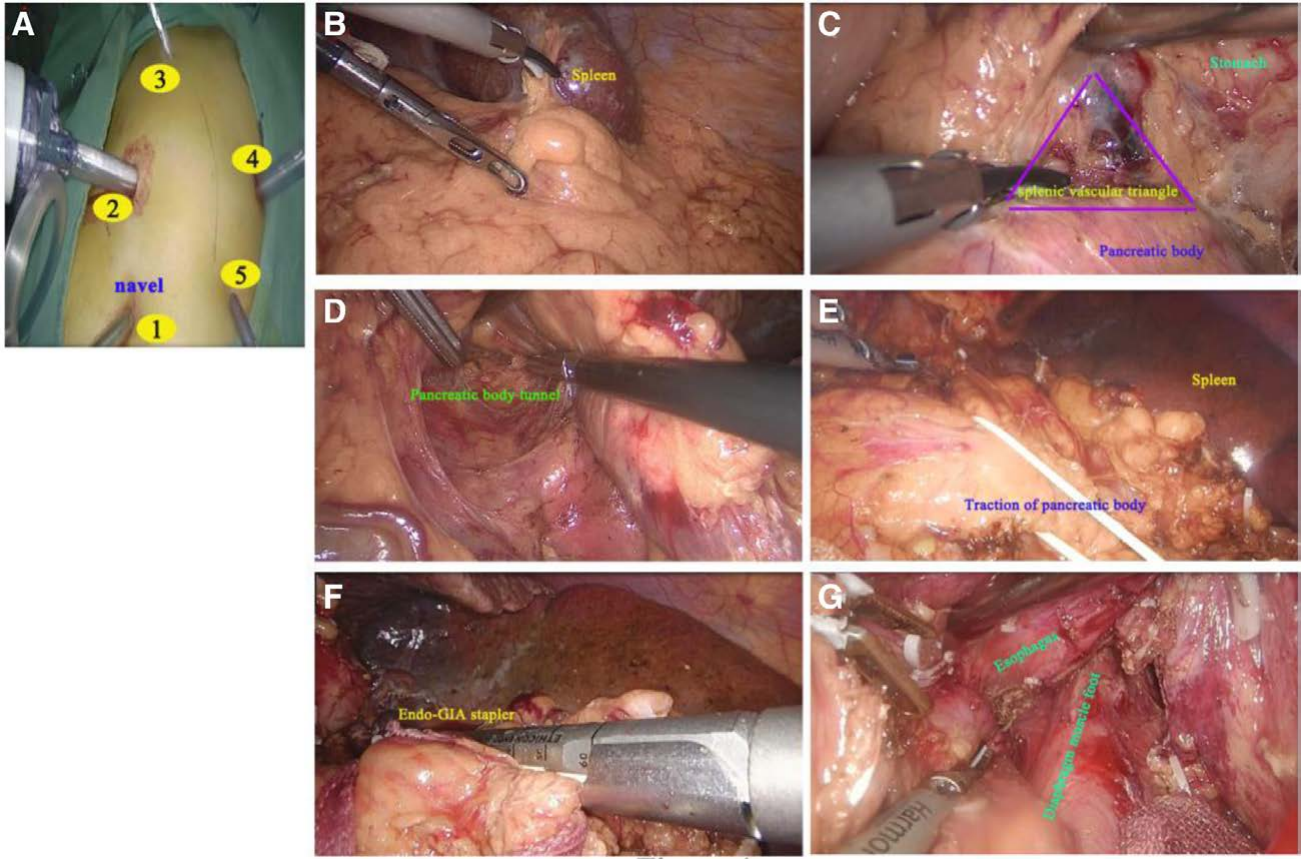
(A) The laparoscopic trocar layout. The 5-hole method with the camera port placed infraumbilically is shown. (B) Dissecting the inferior pole of the spleen. (C) Dissecting the splenic vascular triangle (indicated by the arrow). (D) Establishment of a tunnel behind the pancreatic body to connect with the splenic vascular triangle. (E) The pancreatic body was suspended and pulled caudally exposing the vessels in the upper pole of the spleen for dissection and separation. (F) The splenic portal vessel was divided using a cutting and closure device. (G) Seven cm of “bare” esophagus from the cardiac peripheral blood vessels.

Briefly, the procedure was performed as follows: the patient was positioned in the split-leg herringbone position, gastric and urinary tubes were inserted and routine skin disinfection and draping were performed. The abdominal operation was performed using a 5 trocar method (Fig. 1A). The gastrocolic ligament was divided using a harmonic scalpel. The splenic flexure and the lower pole of spleen were exposed, the planes were divided along the greater curvature of the stomach, and the left gastroomental and the short gastric vessels were ligated. The splenic artery was ligated where appropriate and the tunnel behind the splenic hilum was dissected. A vascular stapler was used to divide the splenic pedicle vessels, then dissociate the pericardial pleneal band, remove the spleen, lift the stomach again and dissociate the pericardial blood vessels along the greater curvature. Attention was then turned to the lesser curvature of the stomach, the pericardial blood vessels along the left gastric artery and vein were divided leaving the lower esophageal (7 cm) and pericardial blood vessels completely bare. Close attention was paid to protecting the vagus nerve and the main artery and vein of the left stomach. Finally, 20Fr latex drains were placed in the posterior stomach and splenic fossa, respectively. Postoperative fasting, gastrointestinal decompression, hemostasis and other treatments were provided. The amylase level was measured on postoperative days 1, 3, and 5.

### 3.2. PBS technique

A Harmonic scalpel was used to divide the gastrocolic ligament to expose the splenic flexure of the transverse colon and the lower pole of the spleen (Fig. 1B). The planes were divided along the greater curvature of the stomach, exposing the upper margin of the pancreas and opening the splenic arteriovenous triangle (the triangle constructed by the upper margin of the pancreatic body, the left gastric artery and the posterior wall of the stomach) (Fig. 1C). Dissection was continued along the lower margin of the pancreatic body to open a tunnel behind the pancreatic body (Fig. 1D). The pancreas was transfixed using a homemade suspension belt of 6 to 8 cm fashioned from a sterile medical glove which was inserted through the splenic arteriovenous triangle from the tunnel of pancreatic body (Fig. 1E). Under the traction of the suspension belt, the fully exposed the short gastric vessels were individually divided. The tunnel behind the splenic hilum was extended, and the splenic pedicle was divided using an Endo-GIA stapler(Ethicon, Johnson & Johnson, USA) (Fig. 1F). The splenic pleneal band was dissociated and the spleen was removed. The selective pericardial devascularization part of the operation was performed (Fig. 1G). Finally, 1 drain was placed behind the stomach and 1 in the splenic fossa.

## 4. Results

### 4.1. Comparison of preoperative data

Eleven patients underwent laparoscopic splenectomy combined with pericardial dissection using the traditional approach. This cohort included 8 males and 3 females with a mean age of 54.55 years and body mass index (BMI) of 20.55. The PBS technique was used in 5 patients. This cohort included 3 males and 2 females with a mean age of 54.2 years and BMI of 21.64. There was no significant difference in age, sex, or BMI between the 2 groups (*P* > .05). There was no significant difference in preoperative WBC, HB, PLT, ALB, prothrombin time, TBIL, Child–Pugh grading, ASA grading, preoperative diagnosis or splenic size between the 2 groups (*P* > .05) (Table 1).

**Table 1:** The comparison of 2 methods for laparoscopic splenectomy with pericardial dissection.

	The traditional approach (n = 11)	Pancreatic body suspension technique (n = 5)	*F/Z*	*P*
*Preoperative*				
Age(yr), mean ± SD	54.55 ± 8.38	54.20 ± 13.85	0.004	.95
Gender			0.382	.54
Male, n (%)	8 (72.73)	3 (60)		
Female, n (%)	3 (27.27)	2 (40)		
BMI (kg/m^2^), mean ± SD	20.55 ± 1.21	21.64 ± 0.84	3.280	.09
ASA, n (%)			1.173	.28
II	7 (63.64%)	2 (40%)		
III	4 (36.36%)	3 (60%)		
WBC (×10^9^/L), mean ± SD	2.78 ± 1.20	2.16 ± 1.15	0.946	.35
HB (g/L), mean ± SD	93.91 ± 27.86	90.60 ± 24.90	0.051	.82
PLT (×10^9^/L), mean ± SD	38.91 ± 13.72	42.20 ± 21.55	0.139	.71
ALB (g/L), mean ± SD	35.05 ± 5.31	34.00 ± 5.03	0.137	.72
PT (s)*	13.8 (12.8–14.7)	15.3 (12.95–18.6)	−1.628	.10
TBIL (μmol/L), mean ± SD	23.82 ± 13.31	22.14 ± 10.43	0.061	.81
Child–Pugh score, n			1.157	.56
A	5	3		
B	5	2		
C	1	0		
Spleen size (cm), mean ± SD	20.06 ± 6.66	19.40 ± 3.78	0.042	.84
Causes of liver cirrhosis, n (%)			4.047	.13
Hepatitis B	8	3		
Alcohol	2	0		
Schistosomiasis	1	2		
*Intraoperative*				
Operative time (min), mean ± SD	240.91 ± 70.87	280.00 ± 84.26	0.935	.35
Estimated blood loss (mL), mean ± SD	290.91 ± 248.82	250.00 ± 239.79	0.095	.76
Conversion to open surgery, n (%)	2 (18.18%)	0 (0%)	2.010	.16
Postoperative				
Discharge with drainage tube (%)	7 (63.64%)	1 (20%)	1.173	.28
Postoperative LOS (d), mean ± SD	8.64 ± 2.06	11.20 ± 1.30	6.410	.02
Complications	4 (36.36%)	0 (0%)	4.492	.03
Bleeding, n (%)	3 (27.27%)	0 (0%)	3.182	.07
Reoperation	1 (9.09%)	0 (0%)	0.955	.33

ALB = albumin, BMI = body mass index, HB = hemoglobin, LOS = length of hospital, PLT = platelet, PT = prothrombin time, TBIL = total bilirubin, WBC = white blood cell.

* Data were shown as median and interquartile range.

### 4.2. Comparison of operative data

The mean operation duration was 240.91 minutes in the traditional group versus 280 minutes in the PBS group (*P* > .05). The mean intraoperative blood loss was estimated to be 290.91 mL in the traditional method group and 250 mL in the PBS group (*P* > .05) (Table 1). There were 2 cases of laparotomy because of bleeding in the short gastric veins and splenic pedicle, respectively. Both were in the traditional method group.

### 4.3. Postoperative recovery and postoperative complications

There was no postoperative death or pancreatic fistula in either group. Seven patients in the traditional method group were discharged with a drainage tube due to a peritoneal effusion with a mean postoperative hospital stay of 8.64 days. One patient in the PBS group experienced a peritoneal effusion with a postoperative hospital stay of 11.2 days, which was significantly longer than that in the traditional method group (*P* > .05). There were 3 cases of postoperative abdominal hemorrhage and 1 case of a return to the operating room in the traditional method group only. The incidence of postoperative complications (postoperative bleeding + reoperation) was significantly higher in the traditional method group than in the PBS group (36.36% vs 0, *P* = .034) (Table 1).

## 5. Discussion

Laparoscopic splenectomy combined with pericardial dissection is a complicated and challenging operation. Traditional open splenectomy combined with pericardial dissection can result in difficulty with subsequent liver transplantation due to intra-abdominal adhesions. Therefore, many surgeons recommend laparoscopic surgery.^[2–9,11–14]^ Patients undergoing this procedure often require surgery for splenomegaly and portal hypertension secondary to cirrhosis. An enlarged spleen can cause blood loss due to reduced platelets. Additionally, portal hypertension can cause bleeding due to ruptured varices in the esophagus and stomach. Thus, this surgery can address both life-threatening conditions.

A challenging aspect of the operation is the control of bleeding.^[2,14]^ Due to high portal pressure, once intraoperative bleeding occurs, life-threatening intraoperative hypotension, shock and death can occur. The effective control of intraoperative bleeding is the key to the success of this operation. The short gastric vessels are the most prone to bleeding.^[2,14]^ Zhang et al^[2]^ reported an improved laparoscopic splenectomy combined with pericardial dissection technique, which recommended the treatment of the short gastric vessels, making the operation safer with less intraoperative blood loss and faster patient recovery.

After performing a large number of early cases using the traditional method, we focused on the technical exploration of the treatment of intraoperative short gastric vein. We constantly evaluated our technique and finally proposed the application of PBS technology in laparoscopic splenectomy combined with selective pericardial dissection. There were 2 main reasons why this was technically feasible: the splenic vascular triangle, composed of the left blood vessel of the stomach, the upper margin of the pancreas and the posterior wall of the stomach, is a relatively safe area. Opening this triangle clearly reveals the arterial and venous trunk of the spleen; the Toldt space behind the pancreas was extended along the lower margin of the pancreas. This is a very safe surgical route with only loose tissue. It can be easily connected to the splenic vascular triangle.

This technique used theoretical innovations. First, after suspending the pancreas, the blood flow into the spleen was occluded reducing the risk of bleeding. This maneuver also pulled the pancreas caudally to fully expose the stomach, the upper pole of the spleen and the blood vessels behind the stomach, so as to make the operation safer and more reliable. Second, after the pancreatic body was suspended, the structures behind the splenic hilum were fully exposed, and the pedicle vessels of the spleen could be handled in a more controlled fashion.

We performed laparoscopic splenectomy combined with selective pericardial vascular dissection in 5 patients and only used the PBS technique during the operation and achieved satisfactory results. None of the patients underwent laparotomy or developed postoperative pancreatic fistula. All patients were discharged successfully. Of the 11 patients in the traditional method group, 2 were converted to laparotomy, 3 suffered intra-abdominal bleeding, and 1 underwent a second operation to stop bleeding. The incidence of postoperative complications (postoperative bleeding + reoperation) in the traditional method group was significantly higher than that in the PBS group. In addition, we observed that the estimated intraoperative blood loss was 290.91 mL in the conventional method group compared with 250 mL in the PBS group. The conversion rate of laparotomy (18.18%) was higher than that of the PBS group (0), but the differences were not statistically significant, which may be related to the small number of sample cases. The length of postoperative hospital stay in the traditional method group was significantly shorter than that in the PBS group. We speculated that this might be related to the high number of patients discharged with a drain in the traditional method group.

There were some shortcomings in this study. First, this was a retrospective study with bias, including the preferred surgical technique of the surgeon and the selection of the patients. Second, the sample size was small. Therefore, relevant conclusions need to be confirmed by follow-up randomized controlled studies with larger sample sizes.

## 6. Conclusion

The application of PBS technique in laparoscopic splenectomy combined with selective pericardial vascular dissection was safe, reliable and had a low incidence of postoperative complications. Relevant research conclusions still need to be confirmed by multi-center large sample prospective controlled studies.

## Acknowledgments

The project was supported by the Anhui Province Key Research (202004j07020051), Anhui Province Health Commission Key Project (AHWJ2023BAc10049), and Yijishan Hospital Talent and Peak Project (YR202122, GF2019T03, and GF2019G03).

## Author contributions

**Conceptualization:** Bin Liu, Bin Jiang.

**Formal analysis:** Xu Wang.

**Investigation:** Shihang Xi.

**Methodology:** Bin Liu.

**Supervision:** Daohai Qian, Xiaoming Wang.

**Writing – original draft:** Daohai Qian, Bin Liu, Bin Jiang.

**Writing – review & editing:** Daohai Qian, Xiaoming Wang.
